# Portal Vein Thrombosis Might Develop by COVID-19 Infection or Vaccination: A Systematic Review of Case-Report Studies

**DOI:** 10.3389/fmed.2021.794599

**Published:** 2021-12-14

**Authors:** Setare Kheyrandish, Amirhossein Rastgar, Morteza Arab-Zozani, Gholamreza Anani Sarab

**Affiliations:** ^1^Department of Hematology and Blood Banking, School of Paramedical Sciences, Birjand University of Medical Sciences, Birjand, Iran; ^2^Social Determinants of Health Research Center, Birjand University of Medical Sciences, Birjand, Iran; ^3^Cellular and Molecular Research Center, Birjand University of Medical Sciences, Birjand, Iran

**Keywords:** COVID-19, vaccines, liver diseases, portal vein, venous thrombosis, case report

## Abstract

**Background and Objective:** Infection by the novel coronavirus disease 2019 (COVID-19) has been associated with different types of thrombotic complications same as portal vein thrombosis (PVT). However, by emerging vaccines of COVID, the thrombosis did not seem to be concerning anymore. Until new findings showed that, the vaccine of COVID itself can cause PVT.

**Method:** We performed an electronic search in PubMed, Scopus, and Web of Sciences to evaluate the possibility of occurring PVT due to infection and vaccination of COVID-19. The results were reported in a narrative method and categorized into tables.

**Result:** Overall, 40 cases of PVT from 34 studies were reviewed in this article. The prevalence of PVT following COVID-19 was more remarkable in males. However, it was more common in females after vaccinations of COVID-19 in the reviewed cases. Regardless of etiology, 20 of PVT cases reviewed in this article had at least one comorbidity. The most common clinical presentation was abdominal pain (AP). After anticoagulant therapies, most of the patients improved or discharged.

**Conclusion:** As long as the laboratory findings are not appropriate enough to predict PVT, the diagnosis of this complication with whatever underlying reason is challengeable, while rapid diagnosis and treatment of that are vital. Therefore, by providing available data in an organized way, we aimed to prepare the information of infected patients for better and easier future diagnosis of PVT in new cases.

## Highlights

- On the basis of studies we reviewed, PVT following COVID-19 was reported more in males, however; this complication was more mentioned in the females after vaccination of COVID-19.
- Patients with comorbidities were more likely to develop portal vein thrombosis with both underlying reasons.
- Liver CT scan beside laboratory findings were useful solutions in diagnosing this complication.
- We suggest examining more about the underlying mechanism of PVT after vaccination because there was a case mentioned in our study with thrombocytosis so vaccine-induced thrombosis might not be the only mechanism leading to PVT.

## Introduction

The new member of the *Coronaviridae* family has started the novel coronavirus disease 2019 (COVID-19) pandemic, which was first reported in Wuhan, China, appearing as a worldwide health crisis. To date, about 239 million people from 223 different countries in the world have experienced some form of this disease. This dreadful pandemic led to over 4.8 million deaths totally and still, the number keeps on increasing (1–3).

Severe acute respiratory syndrome coronavirus 2 (SARS-CoV-2), as it was named by the WHO, is a single-stranded RNA virus. Different proteins play roles in the structure and function of this virus. One of the most important ones is the spike glycoprotein, the extrinsic crown-shaped construction of the virus, which is the key connector for the fusion of SARS-CoV-2 and human being cells (4).

The clinical presentations of COVID-19 vary depending upon the immune system, gender, and age of the patients. General symptoms including fever, cough, and fatigue are common in many patients, but several complications such as thrombosis, severe respiratory symptoms, heart, kidney, and multiorgan failure are less prevalent (4–6). The incidence rate of some symptoms was determined by a meta-analysis in 50,466 patients, as followed; fever 89.1, cough 72.2, fatigue 42.5, and abnormal CT in 96.6% of the cases (7). COVID-19 is associated with different types of coagulation abnormalities and is shown to be associated with an increased risk of arterial thrombosis and venous thromboembolism (VTE). It can mainly cause, disseminated intravascular coagulation (DIC), VTE, deep vein thrombosis (DVT), portal vein thrombosis (PVT), and other coagulopathies (8).

Portal vein thrombosis is an abnormal rare condition associated with malignancy, liver cirrhosis, and acute abdominal inflammation (9). PVT cases due to non-cirrhotic reasons are scarce. After cirrhosis, myeloproliferative neoplasms, surgery, and inflammatory conditions are three major triggers leading to obstruction in the portal vein (10). This disorder usually occurs when thrombus blocks the portal vein partially or completely. This obstruction of the portal vein is categorized in different ways. Due to Baveno VI criteria, PVT may happen whether because of extra hepatic portal vein obstruction or the intrahepatic one. However, splenic or super mesenteric veins are not involved. Another categorization demonstrates that in the chronic form of PVT, patients generally develop symptoms such as varicose veins and hypersplenism that are associated with portal hypertension. Nevertheless, in the acute form, local and systemic prothrombotic factors are the main reasons for this complication (9, 11). The clinical presentations of acute PVT, show a wide spectrum of asymptomatic indications to severe intestinal ischemia and infarction (12). Several surveys indicated that activation of the coagulation pathway by COVID-19 infection might be due to the inflammatory response of cytokines to virus invasion (13). For example, IL-6 can increase the expression of tissue factor (TF) from mononuclear cells, which may lead to clot formation. Besides this, other inflammatory cytokines such as tumor necrosis factor alpha- α (TNF-α) and IL-1 can play roles in anticoagulant pathway inhibition (8).

After introducing different vaccines of COVID-19 to the world, in addition to PVT following the COVID-19, several people have encountered with PVT after being vaccinated against this virus all around the world.

Since PVT may happen due to several reasons including infectious disease, by reviewing the available data from other articles, we tried to study the PVT, which is either caused by COVID-19 itself, or vaccination while narrating the differences and similarities.

## Methods

### Protocol and Registration

We followed the Preferred Reporting Items for Systematic Reviews and Meta-Analyses (PRISMA) for developing and reporting this article (14).

### Eligibility Criteria

All the case report studies that stated PVT on patients with COVID-19 were included in this study. Every study that reported consequences related to PVT following COVID-19 or all the types of vaccines of COVID-19 was included. Ultrasonography and contrast-enhanced CT are two gold-standard investigations of PVT. All the articles using each of these two diagnostic methods besides the studies which had not mentioned the diagnostic method but been approved and published as a PVT case due to infection of COVID-19 or vaccination were included. All the studies without available English full-text were excluded. Studies that report incomplete data or irrelevant subjects were also excluded.

PICOD:

Population: All patients with COVID-19 or vaccinated people against COVID-19.
Intervention: Not applicable.
Comparison: Not applicable.
Outcome: Portal vein thrombosis.
Design: Case-report studies.

### Information Sources and Search

We did an electronic search of PubMed, Scopus, and Web of Sciences to September 7, 2021, without language restrictions. The whole data extracted with these search term combinations “2019 nCoV” or 2019 nCoV or “2019 novel coronavirus” or COVID-19 or “new coronavirus” or “novel coronavirus” or “SARS-CoV-2” or (Wuhan and coronavirus) or “SARS-CoV” or “2019-nCoV” or “SARS-CoV-2” and (“portal vein thrombosis” or “portal venous thrombosis”).

Besides this, these mesh terms were also searched COVID Vaccine and Neurology, AstraZeneca COVID vaccine, ChAdOx1 nCoV-19 COVID vaccine, AZD1222 COVID vaccine, Janssen COVID vaccine, Johnson & Johnson COVID vaccine, Ad26.COV2 COVID vaccine and “portal vein thrombosis” or “portal venous thrombosis” (Appendix 1).

Then, we merged them in Endnote V.8. All the reference lists from the included studies and relevant systematic reviews were hand-searched for additional studies.

### Study Selection

The duplicate studies were removed and the title, abstract, and full-text of records were screened by two independent reviewers based on pre-mentioned inclusion and exclusion criteria. A third reviewer reviewed the record in case of discrepancy, and disagreement was resolved by consultation.

### Data Collection Process and Data Items

Two independent reviewers extracted and tabulated all the relevant data using a researcher-made checklist. Disagreement was resolved by consensus between all the authors. The data extraction checklist includes items such as author name and year of publication, demographic data, clinical presentation, COVID-19 diagnosis test, clinical manifestations related to PVT (same as fever, APs, etc.), PVT location, treatment, and outcome of the therapy (Table 1). Besides all these, the data of laboratory experiments of the patients were categorized in a separate table (Table 2). As long as not all of these parameters were crucial enough to extract the suitable data from the articles with COVID-19 vaccinated cases, we designed another table with the extra following subheadings: Type of vaccine and diagnostic tests, COVID-19 infection test, number of days until the start of symptoms, and abnormal parameters in laboratory examinations.

**Table 1:** Data extraction table of patients suffering from PVT as a complication of COVID-19.

**References**	**Demographic data**	**Clinical presentation**	**Covid-19 diagnoses**	**PVT diagnostic method**	**Clinical manifestations related to PVT**	**PVT location**	**Treatment and anticoagulant therapy**	**Outcome**
Borazjani et al. (15)	M/26y/asthma, alcohol user, cigarette smoker, and occasionally marijuana user	Dyspnea, decrease in the level of consciousness admission with acute asthma attack	Normal CT/positive (RT-PCR) for SARS-COV2	Abdominopelvic CT with IV contrast	AP and abnormal liver biochemistries	Hypo perfused areas in the posterior segment of the right	Prophylactic doses of heparin before PVT (5,000 IU every 12 h) and oral warfarin when discharged	No feedbacks after the patient discharged
de Barry et al. (16)	F/79/none	Fever, deterioration in the patient's general condition, AP in epigastric area diarrhea and dyspnea	Ground-glass opacity in CT/Negative (RT-PCR)	Enhanced CT-scan	AP in epigastric area	Increase of density in Right portal vein	Thrombolysis and thrombectomy of the upper mesenteric artery	Passed away
Franco-Moreno et al. (17)	M/27/none	Serious colic abdominal discomfort, fever and dry cough during 3 weeks before admission	Bilateral consolidations with ground-glass surrounding in both inferior lobes of the lung	Contrast non-enhancing CT-scan	RUQ Tenderness with negative Murphy's sign	Filling defect within the right branch of portal vein	Enoxaparin 1 mg/kg twice daily- acenocoumarol for 6 months	Improved
Jafari et al. (18)	M/26/controlled asthma	Respiratory pain and tiredness	Multifocal patchy consolidations and bilateral pleural effusions in CT scan/Positive RT-PCR	Contrast- enhanced CT-scan	Severe AP located in the RUQ	In portal phase of CT scan	Intravenous heparin infusion (1,000 U/h)	Improved
La Mura et al. (19)	M/72/ Parkinson's disease, anxious-depressive syndrome, mild vascular dementia	Fever, jaundice, and obnubilation	Not reported	Contrast- enhanced CT-scan	Mild AP with bloating and constipation followed by periumbilical tenderness with no rebound reaction nor ascites	Occlusion of the left portal venous system and the secondary branches of the right portal vein	Enoxaparin before PVT diagnosis at 4,000 IU o.d. and after PVT diagnosis increased to 100 IU/Kg b.i.d	Improved
Low et al. (20)	M/51/lower limb DVT	Blood vomiting, respiratory failure	–/–	CT-scan	–	Right and left portal thrombosis and portal vein gas	Intravenous heparin	Improved with no residual portal vein thrombosis
Malik et al. (21)	M/32/obesity and hypothyroid	Hematemesis preceded by fever and cough	Serology tests	CT-scan	Left upper AP	NM	–	Improved
Ofosu et al. (22)	M/55/hyperlipidemia	Fever, dyspnea, altered mental state	Positive PCR/ ground glass opacity main right portal vein	Computer tomography angiography	–	Right portal vein	–	Passed away
Rokkam et al. (11)	F/66/fibromyalgia, gastroesophageal reflux disorder, brain injury due to trauma, high blood pressure, depression, constipation, and anemia	A 10-day diarrhea and 1-day unstable mental status (no respiratory symptoms related to COVID-19)	RT-PCR	CT-scan	mild diffuse tenderness on palpation in abdomen	Left branch of portal vein	Apixaban (5 mg b.i.d)	Improved
Abeysekera et al. (23)	M/42/controlled hepatitis B	Fever, oliguric renal failure, supratherapeutic tacrolimus levels, hyponatremia and beside chest discomfort	–	Abdominal ultrasound and contrast-enhanced CT-scan	AP and constant pain in right hypochondrium	Entire portal vein	Apixaban 5 mg two times per day for at least 6 months	NM but symptoms disappeared
Kolli and Oza (24)	F/44/none	AP and bloating	–	CT-scan	Bloating abdomen with pain in RUQ	–	Heparin, coumadin and vitamin K	NM
Petters et al. (25)	F/3/Liver transplant Recipient with history of Caroli disease,treated hepatic artery thrombosis, PVT, EBV infection	Fever, oliguric renal failure, supratherapeutic tacrolimus levels, hyponatremia	RT-PCR	Ultrasound with doppler	Multisystem inflammatory symptoms and abdominal distention	–	Enoxaparin and tacrolimus	Improved
Sinz et al. (26)	M/38/none	Fever, nausea, diarrhea, coughing and pleural irritation	RT-PCR (Negative) but Detectable SARS-CoV-2 serological antibody	Duplex ultrasound	AP and tenderness in RLQ	Extensive PVT and mesenteric vein stasis	Unfractionated heparin	Improved
Miyazato et al. (27)	M/67/Diabetes/alcohol-related cirrhosis/esophageal varices	Fever, respiratory distress	oxygen saturation test-	Contrast-enhanced CT-scan	–	From superior mesenteric vein to the main trunk of the portal vein	No anticoagulants	–
Sharma et al. (28)	M/28/alcohol user	AP, nausea, vomiting, and constipation	RT-PCR	Contrast-enhanced CT-scan	AP	Extensive PVT and mesenteric vein stasis	LMWH, apixaban	–
Rehman et al. (29)	F/33/none	AP	–	CT abdomen with IV contrast	Acute AP in the RLQ	–	Enoxaparin and warfarin	Improved
Agarwal et al. (30)	F/28/pregnancy	Hypertension and general body swelling	–	Contrast-enhanced CT-scan	AP beside distension and tenderness	–	LMWH, diuretics, beta blockers, terlipressin, and anti-biotic	Improved
Jeilani et al. (31)	M/68/pulmonary disease, Alzheimer's dementia and urinary tract infection	AP, constipation, flatus, umbilical hernia with dry coughs and crepitation in chest	–	CT-scan	AP and constipation	Central filling defect within portal vein	LMWH	Improved
Randhawa et al. (32)	F/62/none	RUQ pain	–	Ultrasound	Pain in RUQ but soft and non-tendered abdomen	Right branch of portal vein	Fondaparinux, spironolactone, warfarin	Improved
Rivera-Alonso et al. (33)	M/51/none	AP in RUQ, fever, discomfort	RT-PCR (Negative) but detectable SARS-CoV-2 serological antibody	Enhanced CT-scan	Pain in RUQ	–	Anticoagulants	Improved
Lari et al. (34)	M/38/none	AP, nausea, vomiting, breath-shortness	–		AP	Extensive PVT	Heparin	In charge

*COVID-19, coronavirus disease 2019; PVT, portal vein thrombosis; AP, abdominal pain; RUQ, right upper quadrant; RLQ, right lower quadrant*.

**Table 2:** Laboratory data table of patients suffering from PVT as a complication of COVID-19.

**References**	**HB (g/dL)**	WBC (*10^**3**^/μL)	PLT (*10^**9**^/L)	**CRP (mg/dL)**	**ALT (U/L)**	**AST (U/L)**	**Bilirubin (mg/dL)**	**D-DIMMER (μg/L)**	**PT(s) or Ratio**	**PTT(s)**	**INR**
Borazjani et al. (15)	14.7	18.1 (12%Lymph)	213	–	67	44	1.41	–	19.2	28	–
de Barry et al. (16)	–	12.6 (lymphopenic)	–	12.5	–	–	–	–	–	–	–
Franco-Moreno et al. (17)	RN	18 (8%lymphocyte)	458	24.5	111	64	-	9.530	RN	RN	RN
Jafari et al. (18)	–	7.2 (lymphosyte39%)	–	9.6	–	–	–	500	39s	–	1.34
La Mura et al. (19)	12.1	4.68	330	2.87	28	-	1.13	5,004	1.02	1.13	-
Low et al. (20)	–	–	–	–	–	–	–	–	–	–	–
Malik et al. (21)	12.5	–	–	–	–	–	–	–	–	–	–
Ofosu et al. (22)	14	9.5	518	3	36	50	0.8	>44	–	–	1.2
Rokkam et al. (11)	10.2	31.9	391	–	12	23	–	–	–	–	1.4
Abeysekera et al. (23)	14.7	13.84	364	4.4	31	–	0.37	–	RN	–	–
Kolli and Oza (24)	–	–	RN	–	–	–	–	RN	RN	RN	RN
Petters et al. (25)	–	–	132	18.9	66	132	0.23	7,822	–	–	–
Sinz et al. (26)	17.2	19.5	281	12.2	RN	RN	1.05	6,870	(PT ratio = 0.67)	56	–
Miyazato et al. (27)	–	–	–	–	–	–	–	7,300	–	–	–
Sharma et al. (28)	13.6	10.4	312	–	86	38	0.8	1,533	–	–	–
Rehman et al. (29)	–	RN	RN	1.45	RN	RN	–	610	RN	RN	RN
Agarwal et al. (30)	13.3	17	93	–	–	–	1.89	3,600	11.1	35.5	0.95
Jeilani et al. (31)	15	12.44	318	30.7	41	–	0.76	894	–	–	–
Randhawa et al. (32)	13.1	RN	–	–	RN	RN	–	RN	RN	RN	RN
Rivera-Alonso et al. (33)	–	21.3	–	5.5	472	577	5	–	–	–	–
Lari et al. (34)	–	Increased	–	–	–	–	–	2,100	–	–	–

A third reviewer rechecked the extracted data.

### Quality Appraisal

All the studies were checked in terms of quality by two independent reviewers using an eight-item Joanna Briggs Institute (JBI) checklist for case report studies. The potential disagreement was resolved by consultation with a third reviewer. This checklist includes eight questions and four-rating score (Yes, No, Unclear, and Not applicable). Each question was scored 1 point for yes, 0 points for unclear and no. Then, studies were categorized as having a high risk of bias if the summary score was 0 to <3, moderate risk of bias if the summary score was between 3 and <6 points, and low risk of bias if the summary score was 6 or higher.

### Synthesis of the Results

Due to potential heterogeneity between studies, we reported the results in a narrative method and categorized them into several items available in Tables 1–3.

**Table 3:** Data extraction table of cases suffering from PVT as a side effect of vaccination of COVID-19.

**References**	**Demographic data**	**Clinical presentation**	**Type of vaccine**	**PVT diagnostic method**	**Days until the start of symptoms**	**PVT location**	**Abnormal parameters in laboratory examinations**	**Treatment and anticoagulant therapy**	**Outcome**
De Michele et al. (35)	Case 1: F/57/mild hypothyroidism and treated breast cancer	Left hemiplegia, right gaze deviation, dysarthria, and left neglect, caused by right middle cerebral artery (MCA) occlusion	ChAdOx1 nCoV-19 vaccine (AstraZeneca)	CT-scan	8	Extensive pulmonary artery and portal vein thrombosis	Low platelet count (from 44 to 23 × 10^9^/L)- low Hb levels (5.4 g/dL)- increased levels of Factor VIII while decreased levels of Factor XIII- high levels of PF4–polyanion complexes pan Ab	Thrombectomy, IVIG, plasma exchange, fondaparinux (after increasing of platelet count)	Hospitalized at critical condition
	Case 2: F/55/ mild hypothyroidism	AP and after several days general seizures and coma	ChAdOx1 nCoV-19 vaccine (AstraZeneca)	CT-scan	7	Extensive portal vein thrombosis with occlusion of the left Intrahepatic branches	Elevated D-dimmer (5,441 μg/L)- decreasing thrombocytopenia (from 133 to 59 × 10^9^/L)- increased levels of Factor VIII	IVIG and dexamethasone	Passed away
Kulkarni et al. (36)	M/46/Buddchiary and MPD	Severe AP	ChAdOx1 nCoV-19 vaccine (AstraZeneca)	Contrast-enhanced	7	–	High level of INR (1.7)- negative anti PF4 Ab	Thrombolysis plus venoplasty, LMWH and dabigatran	Discharged
Sorensen et al. (37)	F/30/ migraine	Headache and ecchimose	ChAdOx1 nCoV-19 vaccine (AstraZeneca)	Duplex ultrasonography and CT-scan	8	–	Low platelet count (51 × 10^9^/L) -low levels of fibrinogen, high D-dimer, and marginally increased ALT- increased levels of Factor VIII and VWF-anti PF-4 Ab positive	Tinzaparin 4,500 IU, fibrinogen, fondaparinux, rivaroxaban	Discharged
Öcal et al. (38)	M/41/none	Headache and AP	ChAdOx1 nCoV-19 vaccine (AstraZeneca)	CT-scan	11	Entire portal vein	Thrombocytopenia (64 × 10^9^/L) and increased D-dimer (42 028 μg/L)- anti PF-4 AB positive	Apixaban, IVIG, argatroban,	NM
Greinacher et al. (39)	F/49/none	Chills, fever, nausea, and epigastric discomfort	ChAdOx1 nCoV-19 vaccine (AstraZeneca)	CT-scan	5	–	Thrombocytopenia (18 × 10^9^/L)- high levels of D-dimer (35,000 μg /L)- elevated amounts of CRP and γGT	IVIG, analgesia, enoxaparin, UFH, prothrombin complex concentrates, and recombinant factor VIIa	Passed away
Graf et al. (40)	M/29/NM	Headache, AP and hematomesis	ChAdOx1 nCoV-19 vaccine (AstraZeneca)	CT angiography	9	Extensive PVT	Thrombocytopenia (32 × 10^9^/L), anti PF-4 Ab positive	IVIG, argatroban,	Improved
Scully et al. (41)	Case 1: F/30/ NM	–	ChAdOx1 nCoV-19 vaccine (AstraZeneca)	–	13	–	Thrombocytopenia (27 × 10^9^/L)- elevated D-dimmer (16,280 μg/L)- anti PF-4 Ab negative	–	Alive
	Case 2: F/55/ NM	–	ChAdOx1 nCoV-19 vaccine (AstraZeneca)	–	6	–	Thrombocytopenia (11 × 10^9^/L)- elevated D-dimmer (26,689 μg /L)	–	Passed away
	Case 3: M/54/NM	–	ChAdOx1 nCoV-19 vaccine (AstraZeneca)	–	10	–	Elevated PT (13.5s) D-dimmer (80,000 μg/L)	–	Passed away
D'Agostino et al. (42)	F/54/Meniere's disease	–	ChAdOx1 nCoV-19 vaccine (AstraZeneca)	Angio-CT and contrast-CT-scan	12	left portal branch	Elevated D-dimer, normocytic anemia (HB 8.7 g/dL), thrombocytopenia and signs of DIC	–	–
See et al. (43)	Case 1: F/between 18-39/NM	Headache, nausea, Muscle pain, chills, fever, AP, and bloating	Janssen (Johnson & Johnson) ad26.cov2.s	Ultrasound	8	–	Mild thrombocytopenia (127 × 10^9^/L), elevated D-dimmer (5,450 μg /L) - anti PF-4 Ab positive	–	Discharged
	Case 2: F/more than 40/NM	Back pain, bruising, AP, fever		Ultrasound	13	–	Thrombocytopenia (13 × 10^9^/L), elevated D-dimmer (112,070 μg /L) – decreased fibrinogen (59 mg/dL)–anti PF-4 Ab positive	–	Not discharged
Aladdin et al. (44)	F/36/NM	Fever, vomiting, and severe headache	ChAdOx1 nCoV-19 vaccine (AstraZeneca)	CT-scan	–	Extensive portal vein thrombosis	Elevated WBC (18.7) (mainly neutrophils), low HB at 10.4 g/dL, and clumped platelets- mild elevated liver enzyme-prolonged PT (45 s), PTT (98 s), INR (4.1) -elevated D-dimer (more than 35,000 μg /L)	Enoxaparin	Passed away
Graca et al. (45)	F/62/obesity, asthma and rhinitis	Fever, AP, vomiting, abdominal tenderness	ChAdOx1 nCoV-19 vaccine (AstraZeneca)	Abdominal CT angiography (CTA)	1 (28 days till the PVT occurred)	Left branch of the portal vein	Anemia (HB 7 g/L), thrombocytosis (780 × 10^9^/L), leukocytosis 13 × 10^3^/μL, elevated CRP (31.07 mg/dL), slightly increased levels of liver enzymes (AST 36 U/L, ALP 126 U/L, GGT 72 U/L, LDH 441 U/L, total bilirubin 1.3 mg/dL	LMWH and endoxaban	Discharged
Umbrello et al. (46)	F/ 36/none	Fever, AP, asthenia and osteoarticular pain	ChAdOx1 nCoV-19 vaccine (AstraZeneca)	Contrast-enhanced CT-scan	17	Complete thrombosis of portal vein	Mild thrombocytopenia (133 × 10^9^/L), anti PF4 antibody pos	UFH, IVIG, and argatroban, apixaban	Stable condition
Ciccone et al. (47)	Case 1: F/35/ OCP user	Headache, nausea and vomiting	ChAdOx1 nCoV-19 vaccine (AstraZeneca)	–	6	–	Thrombocytopenia (44 × 10^9^/L)- elevated D-dimmer level (>8,000 μg /L)	Mannitol, metil prednisolone, fresh plasma, enoxaparin, plasmapheresis	In coma
	Case 2: F/54/none	Headache and vomiting	ChAdOx1 nCoV-19 vaccine (AstraZeneca)	–	2	–	Thrombocytopenia (13 × 10^9^/L)- elevated D-dimmer level (78,254 μg /L)	Enoxaparin, fondaparinux, dexamethasone	Passed away
	Case 3: F/55/none	Headache and fever	ChAdOx1 nCoV-19 vaccine (AstraZeneca)	–	6	–	Thrombocytopenia (31 × 10^9^/L)- elevated D-dimmer level (>10,000 μg /L)	Fondaparinux, metil-prednisolone, mannitol, craniectomy	In coma

*NM, not mentioned; AP, abdominal pain; γGT, γ-glutamyltransferase; ALT, alanine transaminase; AST, aspartate aminotransferase; LDH, lactate dehydrogenase; CRP, C-reactive protein; IVIG, intravenous immunoglobulin; WBC, white blood cell; HB, hemoglobin; UFH, unfractionated heparin; LMWH, low-molecular-weight heparin; RN, reported as normal; Ab, antibody*.

## Results

### Literature Search

**Figure 1:**
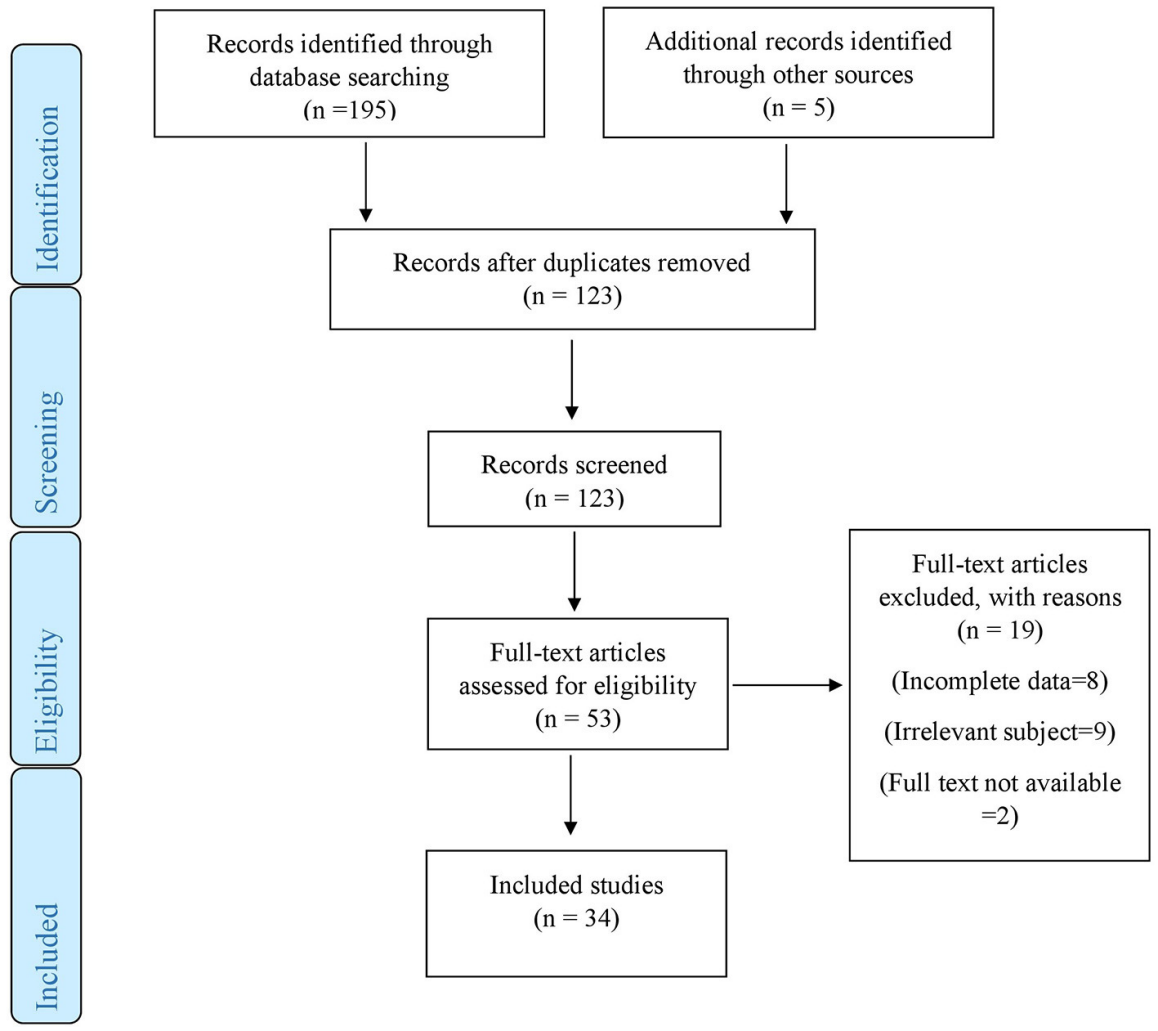
PRISMA flowchart.

The initial search produced 200 articles from three main databases. After removing duplicates, 123 articles remained. These 123 articles were evaluated based on title and abstract and eventually, 53 articles were selected. The full text of these 53 articles was assessed for eligibility criteria. Finally, 34 studies with 40 cases reported the incidence of PVT resulting from COVID-19 (21 cases) or vaccination of COVID-19 (19 cases) due to our inclusion criteria and 19 studies were excluded due to incomplete data, irrelevant subject, or non-availability of full text (Figure 1).

### Study Characteristics and Demographic Data

The characteristics of the included studies are summarized in the three tables. All of the 34 studies were case reports. The data about patients with the PVT following COVID demonstrates a wide age range of 3–79 years. About 66.7% of 21 cases were male. Thirteen of twenty-one cases (61.9%) had at least one comorbidity. There were five cases with liver disorders (four cases with alcohol consumption and one case with controlled hepatitis) three cases with brain injuries, two cases with asthma, two cases of thrombosis, and one case with pregnancy.

However, these amounts differed in PVT following vaccination. The median age of these cases was about 45 years (range 29–62 years) (the age of two cases were reported as ranges, so they were ignored in calculating median age). Only four cases were male and about 79% of the cases were female. Seven of nineteen cases had the previous history of the disease. Hypothyroidism was reported in two cases, Budd-Chiari syndrome and myeloproliferative disorder (MPD) were reported in one case at the same time. Other previous diseases such as migraine, asthma, Meniere's disease, and oral contraceptive pill (OCP) use were each reported in one separate case. Five had no known comorbidities and in seven cases, it was not mentioned. All of the cases of this complication had received the AstraZeneca vaccine except two of whom were injected with Johnson & Johnson.

### Quality Assessment

The JBI tool for quality assessment of included studies yielded scores ranging from 4 to 7. Mean methodological quality was 6.06 out of 8. A total of 31 studies were classified as low risk of bias (77.5%) and 9 studies were with moderate risk of bias (22.5%). Details of the answers to the 8 questions of the tools are given in Appendix 2.

### Clinical Presentation

As was expected, almost different presentations were observed in PVT following COVID and vaccines of COVID. After infecting with COVID-19, 14 cases presented the APs probably because of their PVT. Ten cases presented fever, which is one of the most common symptoms of both the virus and PVT. Seven cases had respiratory problems. Nausea and vomiting were observed in five cases. Four cases complained from cough and the same number of cases were involved with mental problems. Other presentations same as diarrhea, jaundice, and hypertension were presented in fewer cases.

As the same, the AP was a prevailing manifestation in most cases (eight cases) of PVT following vaccination, especially at the right upper quarter (RUQ), where the liver is located. Then, headache (eight cases) and fever (seven cases) were the most prevalent ones, respectively. Nausea and vomiting were presented in the eight cases. Ecchymosis, chills, and muscle pains were less common.

### Laboratory Indices

The most remarkable point about laboratory tests in COVID-19 infected patients was the high level of CRP in all the cases that this index was measured (it was measured in 11 cases).

Only two out of twenty-one patients were associated with abnormal hemoglobin (Hb) levels. Variable platelet counts (PLT) were reported in cases (1 case with decreased, 1 case with increased, and 11 cases with normal counts of PLTs). White blood cell (WBC) counts never dropped under the normal range. Among the cases manifesting PVT following COVID-19, nearly half of the patients (11 cases) appeared with leukocytosis. Liver enzymes were normal (5 cases) to elevated (9 cases) as was expected in PVT disease. Bilirubin was elevated only in three cases. The level of D-dimer was elevated remarkably more than normal in nine patients. Coagulation tests were not performed for most of the patients and did not show a significant increase in performed cases (PT, PTT, and INR were normal in 7, 6, and 5 cases, respectively, and were only elevated in 2 cases).

The results of laboratory experiments in PVT following vaccination showed different algorithms. Thrombocytopenia was a prevalent finding in most of the cases. Fifteen out of nineteen (79%) had experienced low-platelet counts. However, one case was reported with thrombocytosis. The D-dimer level was elevated in 14 cases as a sign of thrombosis. Anti-PF4 antibody was positive in seven cases and negative in two cases. Coagulation tests were abnormal in nine cases. Other complementary data are given in Table 3 (Only abnormal indices are reported).

### Treatments and Outcomes

To treat PVT following COVID-19, different drugs were prescribed depending upon the condition of the patient. Enoxaparin was the most commonly utilized treatment and fortunately, most cases improved after treating it. Six cases used heparin. Moreover, the same number of cases (3) were treated with warfarin or Apixaban. Although in several cases, heparin or enoxaparin were prescribed as prophylaxis therapy before the occurrence of PVT and after the diagnosis COVID-19, PVT developed in some cases. In 1 case, thrombectomy was performed, but finally the patient passed away. Most of these drugs led to improving or discharging the patient. There were only two cases with not clear outcomes and two cases who were passed away.

Nevertheless, when PVT was presented after vaccination the most utilized drugs were intravenous immunoglobulin (IVIG) and low molecular weight heparin (LMWH). Argatroban, Apixaban, unfractionated heparin, thrombectomy, and thrombolysis were the other more prevalent treatments used, respectively. From the postvaccination PVT, six cases passed away, five cases were reported as still in charge, and six cases were discharged (the outcomes of two patients were not mentioned).

## Discussion

**Figure 2:**
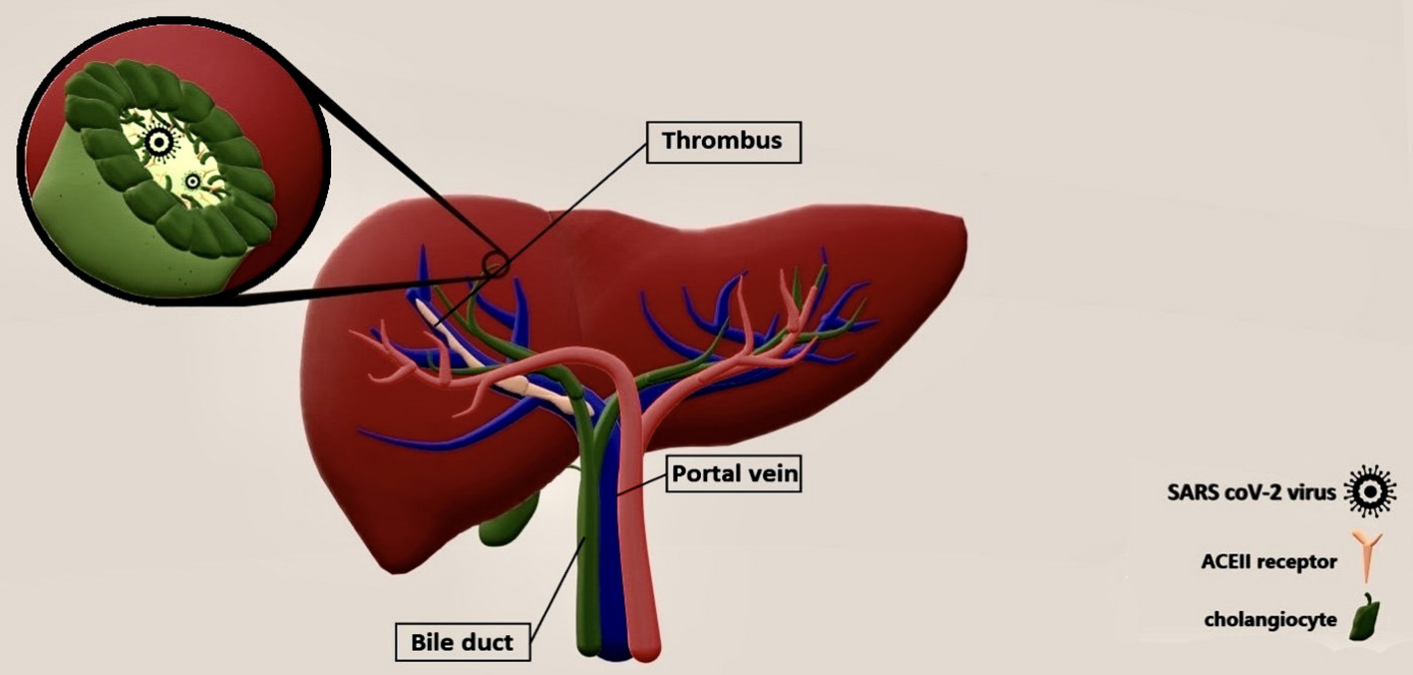
Possible underlying mechanism of PVT by COVID-19 infection. Cholangiocyte is a kind of liver cell which has ACE II receptors presented on the surface more than hepatocytes and endothelium. By direct fusing of COVID-19 to these cells, first the direct injury of the liver happens because of the accumulation of bile acids. Then several inflammatory cytokines (IL6, TNF-alpha) are secreted and they play as inflammation and thrombosis triggers in the liver. Clot formation in the portal vein might be because of the increased expression of tissue factor (TF) from mononuclear cells, possibly done by IL-6. Beside this, other inflammatory cytokines such as tumor necrosis factor alpha (TNF-alpha) and IL-1 can play roles in anticoagulant pathway inhibition.

**Figure 3:**
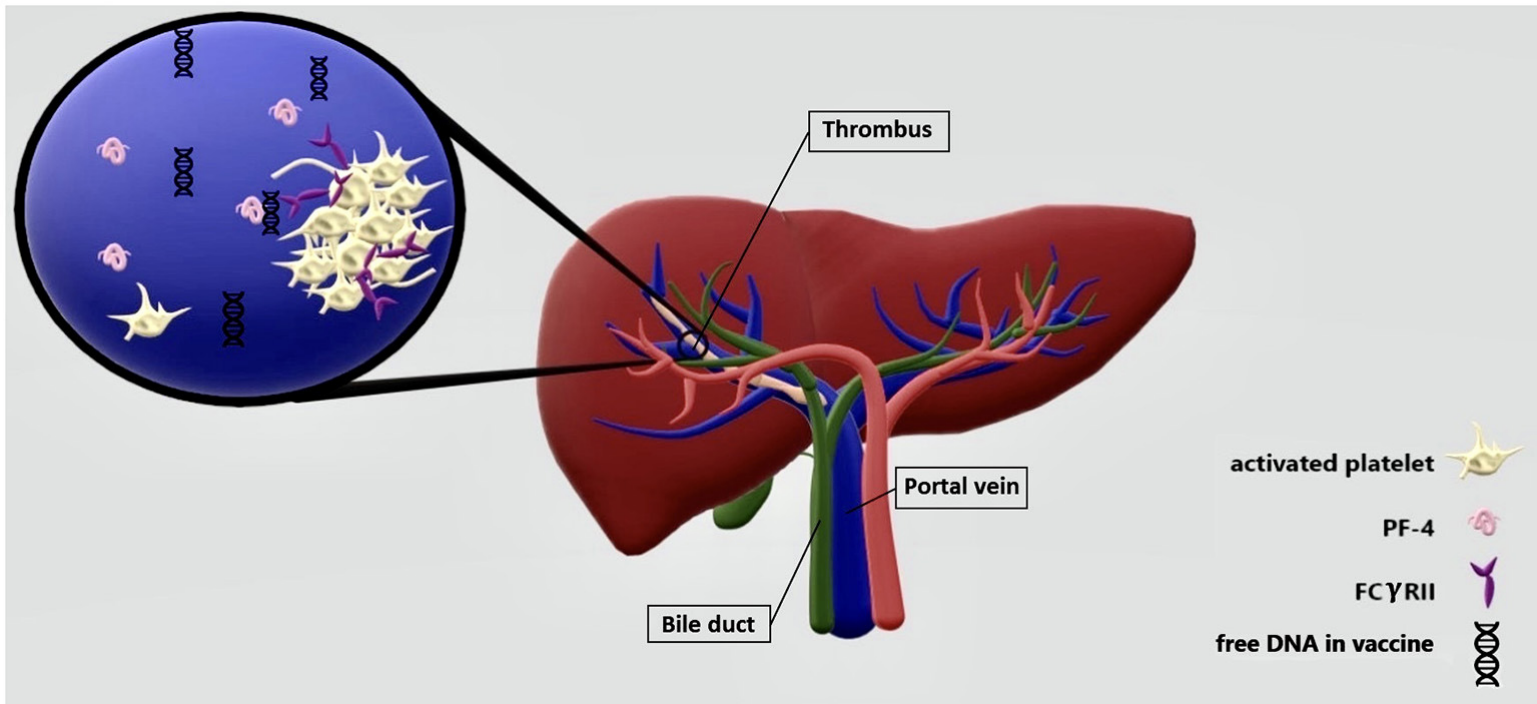
Similar to HIT, vaccine induced thrombocytopenia and thrombosis (VITI) occurs because of free DNA available in COVID-19 vaccines. The free DNA stimulates the production of PF-4 molecules from platelets. The PF4-free DNA attaches to FCRYll on the surface of platelets and the platelet clot shapes.

In this study, we review 40 cases with PVT because of new etiologies, the COVID and the vaccines of COVID-19. In this study, the median age was 41 years with the preference sex of males in cases of COVID. However, in PVT following vaccination, the median age of cases was 45 and 79% of them were females. The average number of days until onset of the symptoms was 8.3 days. As long as this is a systematic review of the cases, it is only possible to narrate the existed data. For comparisons, a cohort study is suggested on two homogenate groups. The underlying mechanism of PVT following COVID-19 is not precisely clear but Marjot et al. declared that the attachment of the virus might occur through ACE-2 receptors on the surface of cholangiocytes so the local presentation of COVID-19 in the liver, makes the body to produce different cytokines against it, which leads to the liver injury (Figure 2) (48). As portal vein is a vital part of the liver, the thrombosis may be formed because of the same reason. In addition, there is another theory provided by Mohseni Afshar et al. suggesting a mechanism for vaccine-induced thrombosis (VIT). They recommended that thrombi are formed in the vessels dependent or independent of heparin. As long as most of the vaccinated cases were non-heparin users, this heparin-induced thrombocytopenia (HIT) may be happened in a spontaneous or autoimmune way (called aHIT). In aHIT, it is not necessary for heparin to be present and other reasons same as free DNA do job of heparin. The free DNA attaches to PF4, and then platelets to make thrombus (49). The positive anti-PF4 antibody in seven out of nine reviewed cases somehow confirmed these findings. Almost in every type of HIT, the decreased counts of platelets are supposed to observe (Figure 3). However, in a 62-year-old woman with PVT that was reported by Graca (mentioned in Table 1), the opposite happened. The case was an asthmatic patient with the thrombocytosis of 780 × 10^3^ per milliliter (45). Therefore, still further studies are needed to figure out other underlying mechanisms of the PVT following vaccination and solve this paradox. A recent cohort study stated the incidence of PVT following COVID-19 was 392.3 per million people, which was significantly higher than in PVT following vaccination (AstraZeneca and Jansen vaccines were excluded from this study) (49, 50).

Chronic PVT is a persistent obstruction of the portal vein often more than 6 months from the onset of the presentation. Therefore, the mentioned data suggest that PVT following infection of COVID-19 is of acute form. In PVT, the clinical presentation of the patients are as followed: AP (61%), hepatomegaly (67%), and ascites (83%) while about 20% of the patients have no symptoms (10). Data presented in this study demonstrated that in PVT following both etiologies, the AP was the most prevalent presentation whether after COVID infection or vaccination (66% after COVID-19 and 42% after vaccination).

Examinations of available data demonstrated that laboratory indices are not proper assistances in confirmed diagnosis or prognosis of PVT, and ultrasonography or contrast-enhanced CT scan of the hepatic portal vein is a better way for a valid diagnosis. The Baveno VI criteria suggest that Doppler ultrasound, CT scan and MRI are the best ways to diagnose both the presence and extension of PVT. However, some laboratory indices will be helpful. The elevation of D-dimer was expected, same as PVT cases due to non-COVID-19 etiologies (51). The liver function tests were normal to elevated because in PVT, the liver retrieves the condition by enhancing hepatic arterial flow (52, 53). In accordance with our result, another study suggested that mild elevation of ALT and AST enzymes are estimated in 29–39% and 38–63 % of patients of COVID, respectively (48). There was also a study, which mentioned that in cases of PVT, evaluation of mean platelet volume (MPV) would help as a diagnostic index. They declared that the larger the platelets, the higher the thrombotic conditions (54). However, the MPV amount was not mentioned in any of the studies we examined. As long as PVT is an uncommon complication without pathognomonic clinical manifestations, the mentioned laboratory indices beside general clinical presentations such as fever and AP are helpful in diagnosis of PVT in COVID-19 infected patients. Moreover, these parameters shift to AP and headache beside thrombocytopenia, D-dimer levels, coagulation tests, and anti-PF4 antibody detection in the cases who are suspected to PVT following vaccination.

Up to now, anticoagulants are the suggested treatments for PVT. Due to presence of hypercoagulable states, the treatment can be considered long term or short term. Heparin and low-molecular-weight heparin (such as enoxaparin) was prescribed in more than half of the cases. Nearly all of them were improved except 2 with uncertain feedbacks. While most of the studies suggest these 2 drugs for solving the thrombosis problem in COVID-19 infected patients, there is one case report that advises platelet count monitoring due the probable risk of HIT. In addition, because the similar mechanism (aHIT) underlies the PVT related vaccination of COVID-19, IVIG, LMWH, and fondaparinux were the most utilized drugs for patients with this trouble. Two of COVID-19 infected and six of vaccinated cases were passed away. Due to heterogeneity of the age, sex, and comorbidities of the patients it is not possible to report which condition is associated with less mortality but due to other studies it is clear that vaccine is safe (55) and the mortality rate of thrombosis is much lower than the infection itself. However, in general, the thrombosis morbidity rate is less after vaccination and even when it is occurred, the condition is manageable. Nevertheless, as long as HIT-like mechanisms are suggested as the main responsible of PVT occurrence after vaccination, the use of heparin is with more caution and the wide-spectrum of the utilized drugs somehow confirms this challengeable condition.

However, we were confronted with several limitations same as small numbers of studies and lack of strong evidence, so further examinations are needed in future studies for more information.

## Conclusion

In this systematic review, we have tried to prepare data available on two new etiologies of acute PVT. Even if the patient is receiving anticoagulants as prophylaxis therapy of PVT, this complication might happen after infection or vaccination of COVID-19. Therefore, it is recommended that upon observing the clinical symptoms mentioned (the most important one is ap), provide a liver CT scan for the patient for checking whether the thrombosis involved this vein or not.

Same as infection of COVID-19, the morbidity rate was higher in male PVT cases after infection with the virus. Although the reviewed cases suggested if the PVT was presented due to vaccination, it is more prevalent in females. As it was the most common comorbidity in the presented cases, liver disorders, might had been deteriorated through drug-induced injury, inflammation or anoxia that resulted from COVID-19 (56).

Further studies are needed to exactly clarify that how the virus and the vaccination can lead to thrombosis of portal vein. Moreover, a cohort study is suggested to compare the data and results in two homogenate groups of patients with PVT following COVID-19 and vaccination of COVID-19.

## Data Availability Statement

The original contributions presented in the study are included in the article/Supplementary Material, further inquiries can be directed to the corresponding author.

## Author Contributions

SK and AR contributed in design, acquisition of data, and drafting the manuscript. MA-Z contributed in the interpretation of data and drafting the manuscript. GS was the study supervisor and contributed to all the aspects of the study. All authors contributed to the article and approved the submitted version.

## Conflict of Interest

The authors declare that the research was conducted in the absence of any commercial or financial relationships that could be construed as a potential conflict of interest.

## Publisher's Note

All claims expressed in this article are solely those of the authors and do not necessarily represent those of their affiliated organizations, or those of the publisher, the editors and the reviewers. Any product that may be evaluated in this article, or claim that may be made by its manufacturer, is not guaranteed or endorsed by the publisher.
