# Supplementary material for: Portal Vein Thrombosis Might Develop by COVID-19 Infection or Vaccination: A Systematic Review of Case-Report Studies
Source: Front Med (Lausanne). 2021 Dec 14;8:794599. doi: 10.3389/fmed.2021.794599 (PMC8712467; doi:10.3389/fmed.2021.794599)
Supplement: Supplementary file 1 [file Table_1.DOCX]

**Appendix 1: Full search strategy for databases**

‘2019 nCoV’ or 2019nCoV or ‘2019 novel coronavirus’ or COVID-19 or ‘new coronavirus’ or ‘novel corona-virus’ or ‘SARS CoV-2’ or (Wuhan AND coronavirus)or ‘SARS-CoV’ or ‘2019-nCoV’ or ‘SARS-CoV-2’ or “COVID Vaccine and Neurology” or “AstraZeneca COVID vaccine” or “ChAdOx1 nCoV-19 COVID vaccine” or “AZD1222 COVID vaccine” or “Janssen COVID vaccine” or “Johnson and Johnson COVID vaccine” or “Ad26.COV2 COVID vaccine” and (‘portal vein thrombosis’ or ‘portal venous thrombosis’)

**Appendix 2: Quality appraisal of included studies**

| **number** | **CITATION** | **Q1** | **Q2** | **Q3** | **Q4** | **Q5** | **Q6** | **Q7** | **Q8** | **TOTAL**  **SCORE** |
| --- | --- | --- | --- | --- | --- | --- | --- | --- | --- | --- |
|  | **Borazjani et al. 2020** | Y | Y | Y | Y | Y | Y | U | N | 6 |
|  | **Franco-moreno et al. 2020** | Y | Y | Y | Y | Y | Y | U | Y | 7 |
|  | **Debarry et al. 2020** | Y | N | Y | Y | N | N | N | Y | 4 |
|  | **Jafari et al. 2020** | Y | Y | Y | Y | Y | Y | U | U | 6 |
|  | **Lamura et al. 2020** | Y | Y | Y | Y | Y | Y | N | N | 6 |
|  | **Ofosu et al. 2020** | Y | Y | Y | Y | Y | Y | N | Y | 7 |
|  | **Low et al. 2020** | Y | Y | Y | Y | Y | Y | Y | N | 7 |
|  | **Malik et al. 2020** | Y | Y | Y | Y | Y | Y | N | N | 6 |
|  | **Petters et al. 2021** | Y | Y | Y | Y | Y | Y | Y | N | 6 |
|  | **Abeysekera et al. 2020** | Y | Y | Y | Y | Y | Y | Y | N | 6 |
|  | **Espinoza et al. 2021** | Y | Y | Y | Y | Y | Y | N | N | 5 |
|  | **Rokkam et al. 2021** | Y | Y | Y | Y | Y | Y | Y | N | 6 |
|  | **Sinz et al. 2021** | Y | **U** | Y | Y | Y | Y | N | N | 5 |
|  | **Miyazato et al. 2021** | Y | Y | Y | Y | Y | N | N | N | 5 |
|  | **Sharma et al. 2021** | Y | Y | Y | Y | Y | N | N | N | 5 |
|  | **Kolli et al. 2021** | Y | Y | Y | Y | N | N | N | Y | 5 |
|  | **Rehman et al. 2021** | Y | Y | Y | Y | Y | Y | N | Y | 7 |
|  | **Randhawa et al 2021** | Y | Y | N | Y | Y | Y | N | Y | 6 |
|  | **Jeilani et al 2021** | Y | Y | Y | Y | Y | Y | N | Y | 7 |
|  | **Rivera-Alonso 2021** | Y | N | Y | Y | Y | Y | N | Y | 6 |
|  | **Lari 2021** | Y | Y | Y | Y | Y | Y | N | Y | 7 |
|  | **De Michele 2021** | Y | Y | Y | Y | Y | Y | N | Y | 7 |
|  | **Kulkarni 2021** | Y | Y | Y | Y | Y | Y | N | Y | 7 |
|  | **Sørensen 2021** | Y | Y | Y | Y | Y | Y | N | Y | 7 |
|  | **Öcal 2021** | Y | Y | Y | Y | Y | N | N | Y | 7 |
|  | **Greinacher2021** | Y | Y | Y | Y | Y | Y | N | Y | 7 |
|  | **Graf 2021** | Y | Y | Y | Y | Y | Y | N | Y | 7 |
|  | **Scully 2021** | Y | Y | N | N | N | Y | N | Y | 4 |
|  | **D’Agostino 2021** | Y | Y | N | Y | N | N | N | Y | 4 |
|  | **See 2021** | Y | Y | Y | Y | N | Y | N | Y | 6 |
|  | **Aladdin 2021** | Y | N | Y | Y | Y | Y | N | Y | 6 |
|  | **Graca 2021** | Y | Y | Y | Y | Y | Y | N | Y | 7 |
|  | **Umbrello 2021** | Y | Y | Y | Y | Y | Y | N | Y | 7 |
|  | **Ciccone 2021** | Y | Y | Y | N | Y | Y | N | Y | 6 |

Q1. Were patient’s demographic characteristics clearly described?

Q2. Was the patient’s history clearly described and presented as a timeline?

Q3. Was the current clinical condition of the patient on presentation clearly described?

Q4. Were diagnostic tests or assessment methods and the results clearly described?

Q5. Was the intervention(s) or treatment procedure(s) clearly described?

Q6. Was the post-intervention clinical condition clearly described?

Q7. Were adverse events (harms) or unanticipated events identified and described?

Q8. Does the case report provide takeaway lessons?

Y: Yes N: No U:Unclear NA: Not applicable
